# Does It Pay to Treat Patients With Coronavirus Disease 2019? Social Perception of Physicians Treating Patients With Coronavirus Disease 2019

**DOI:** 10.3389/fpsyg.2021.781220

**Published:** 2022-01-13

**Authors:** Shlomo Hareli, Or David, Fuad Basis, Ursula Hess

**Affiliations:** ^1^School of Business Administration, University of Haifa, Haifa, Israel; ^2^Laboratory for the Study of Social Perception of Emotions, University of Haifa, Haifa, Israel; ^3^Managerial Board, Rambam Health Care Campus, Haifa, Israel; ^4^Technion Faculty of Medicine, Haifa, Israel; ^5^Department of Psychology, Humboldt University of Berlin, Berlin, Germany

**Keywords:** person perception, emotions, COVID-19, social perception, attitudes

## Abstract

During the coronavirus disease 2019 (COVID-19) pandemic, the public has often expressed great appreciation toward medical personnel who were often shown in the media expressing strong emotions about the situation. To examine whether the perception of people on a physician is in fact influenced by whether the physician treats patients with COVID-19 and the emotions they expressed in response to the situation, 454 participants were recruited in May 2020. Participants saw facial expressions of anger, sadness, happiness, and neutrality which supposedly were shown by physicians who were presented as working either in COVID-19 wards or in an internal medicine ward. Participants rated how competent, empathetic, caring, and likable each physician was, to what degree they would wish to be treated by each physician, and what salary each physician deserved. Physicians treating patients with COVID-19 were seen more positively and as deserving higher pay; they appeared more competent, caring, likable, and were more likely to be chosen as a caregiver compared to physicians not treating patients with COVID-19. The expressed emotions of physicians had a strong impact on how they were perceived, yet this effect was largely unrelated to whether they treated patients with COVID-19 or not such that happy physicians seemed more empathetic, caring, and likable than the physicians who showed negative emotions. Positive regard toward physicians treating patients with COVID-19 was associated with the fact that they were seen as saving lives and not due to the risk imposed by their work.

## Introduction

Especially at the beginning of the coronavirus disease 2019 (COVID-19) pandemic, the public expressed great appreciation of medical personnel treating patients with COVID-19. Applauses were seen in many cities, and social media was flooded with messages of support for those braving the outbreak to help others (Health Management Organization, 2020). Reports and stories in the news and social media talked about the burden on hospitals and the long hours that doctors and nurses worked to mitigate inadequate resources (Yong, 2020, November, 13). In addition, they mentioned the risks involved in treating patients with COVID-19 (Jewett et al., 2020; Landauro, 2020) while trying to save the lives of patients (Pandey and Sharma, 2020), which may explain this appreciation. In fact, there is evidence that doctors who treated patients with COVID-19 were at considerable risk, especially at the beginning of the pandemic (Misra, 2020).

Other news reports have presented the emotional responses of physicians to the pandemic. For example, some physicians reacted with anger and sadness to the negligence and refusal of people to recognize the risks of the disease (Jackson, 2020) or showed helplessness when there was little they could do to save the lives of patients (Lakhera, 2020). Such reactions by medical personnel may not be expected by the public as, stereotypically, physicians are considered to be unemotional (Hareli et al., 2013). Yet, given the specific context in which such emotions were expressed during the pandemic, the reactions in question may be seen as an appropriate and even desirable response to the situation as well as an indication of the caring attitude of physicians and as such positively affect the perception of the public on the physicians treating patients with COVID-19.

Against this background, a few questions arise. First, we wanted to assess whether, in fact, physicians treating patients with COVID-19 prior to the availability of a vaccine were perceived more positively than physicians who did not. In particular, news reports tended to imply that the positive public reaction toward medical personnel treating patients with COVID-19 was related to the risk involved in treating these patients as part of the effort to save their lives. Hence, we further assessed the degree to which judgments and attitudes toward medical personnel during the COVID-19 pandemic were due to their perceived exposure to risk and/or the saving of lives.

Another aspect is how the views of participants on the physicians were influenced by the emotions that they expressed as a function of the information that the physician treated patients with COVID-19 or not. As noted earlier, the media featured a number of different emotional reactions by physicians. Yet, emotions signal not only an internal state but also the wider values and resources of a person (Hareli and Hess, 2010). Specifically, emotions arise when something important for the emoter occurs and the specific emotion that is felt reflects how the emoter appraises the situation (Frijda, 1986). According to appraisal theories of emotions (Scherer, 1987), when something desirable to the individual occurs, positive emotion is a more likely reaction. By contrast, when something undesirable occurs, the expected emotion is a negative one. The specific positive or negative emotion experienced will further depend on the appraisals of additional aspects of the situation. For example, when an undesirable situation is appraised to be caused by something that the emoter sees as in their power to handle, anger is the likely response. By contrast, when the undesirable situation seems to be caused by something that the emoter cannot handle, sadness is the more likely response (Scherer, 1987). People have a naïve grasp of what causes specific emotions and often use this knowledge to infer how the emoter perceives the environment (Hareli and Hess, 2010). As such, a person who shows anger should be perceived as more competent than one who shows sadness (Tiedens, 2001). Also, a person expressing sadness is seen as less affiliative than a person expressing happiness but as somewhat more affiliative than a person expressing anger (Knutson, 1996). However, emotional expressions interact with the context in which the emotions are expressed. Thus, the same emotion can be perceived as more or less appropriate in a given context, which influences liking and how close one feels to the expresser (Kastendieck et al., 2020). In fact, the impressions generated by an emotional expression can completely reverse depending on context. Thus, Hareli and Hess (2010) found that a person who expresses anger in the face of perceived injustice is perceived as more warm and caring than someone who remains neutral -- attributions that are normally reversed.

The treatment of patients with COVID-19 may also be such a context. This, because an emotional reaction on the part of physicians treating patients with COVID-19 may seem more appropriate when the emotion shown is linked to their work in an unusual and, in many ways, frustrating and frightening situation rather than when shown by physicians in the context of their usual work. In this vein, the emotions shown by the purported physicians should affect the perception of the physicians depending on their purported task such that anger and sadness are linked to a more positive evaluation when shown by physicians who treat patients with COVID-19 than those who do not.

In short, we predicted that: H1: participants would rate physicians who treated patients with COVID-19 more positively than those who did not. This regards how competent, empathetic, caring, and likable each physician appeared. We further asked whether participants would like to be treated by this physician as another proxy for the perceived competence and empathy of the doctor, and we asked about the proposed salary of the physician, as a proxy for their value to society and we expected more positive responses for physicians who treat patients with COVID-19.

H2: Participants’ ratings of the physician would be affected by the emotions they supposedly showed during an interview regarding their work. We expected this effect to interact with the information of whether they treated patients with COVID-19 (H3) in that anger should generally lead to a more negative judgment but not when the physician treated patients with COVID-19, as in this complex situation, anger can be a signal for caring.

The study reported below was conducted in May 2020 with participants from the United States: that is, data collection was conducted at the height of the first wave of the pandemic and before established treatments and vaccines were available.

## Materials and Methods

### Participants

In total, 454 (226 women, 2 unknown gender) participants with a mean age of 41 years (*SD* = 13.99) were recruited through Amazon MTurk. Based on a sensitivity analysis using G*Power (Faul et al., 2007), given our sample size, the minimum effect size that the experiment had 80% power to detect was *f* = 0.15.

### Stimuli

**FIGURE 1:**
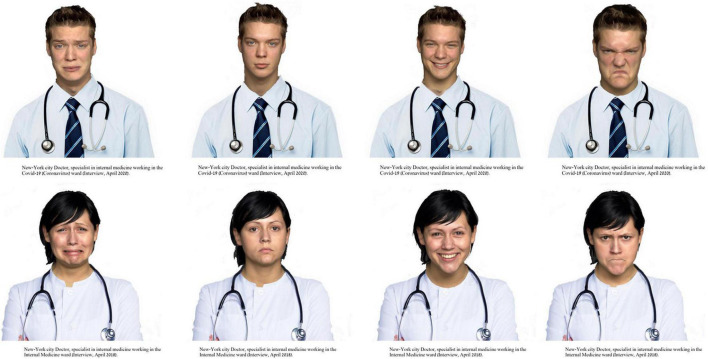
Examples of stimuli used in the study. Images with permission from the FACES Database.

Facial expressions of anger, sadness, happiness, and neutrality by 4 men and 4 women were taken from the FACES set (Ebner et al., 2010), resulting in a total of 4 (emotion) × 2 (gender) × 4 (identities) = 32 faces. Using Photoshop, posers were “dressed” in a white laboratory coat with a stethoscope being shown (see Figure 1 for examples). Participants received the information that they were about to see a photograph of a doctor working in a hospital in New York City and that the photograph was taken during an interview with the doctor about their work. They then saw a photograph of a physician (see example shown in Figure 1). Below the photo was information about the ward the physician supposedly worked in and the date of the interview. Specifically, participants were told that the physician works either in the COVID-19 (Corona Virus) ward or in an internal medicine ward. The interview was described as having taken place in April 2020 or April 2018. This was done in order to keep the descriptions of the doctors as similar as possible except for the fact that one of them treated patients with COVID-19 and the other did not as the earlier interview clearly took place before the outbreak of the pandemic. The information was presented below the photographs. Each participant saw only one picture in a complete between-participants design.

### Dependent Variables

Participants were instructed to describe their impression of the physicians by indicating how competent, empathetic, caring, and likable each physician appeared. Initial results showed that these items were highly correlated (*r* = 0.60–0.91), and hence, we combined them into a single scale (α = 0.92) called positive evaluation.

Participants further indicated to what degree they would wish this doctor would treat them were treatment necessary. Then, participants rated how risky they thought the work of physicians is as well as whether they thought the work of physicians saved lives. All scales were anchored at 0 = not at all and 6 = very much.

Then participants determined the annual salary each physician deserved to be paid, given that average salaries are around US$266,000 annually (salary.com, 2020). The salary scale ranged from US$200,000 to US$350,000 with the slider set initially at the average annual salary (i.e., US$266,000). Using the same answer format as for the person perception scales, participants were asked to report the extent to which the COVID-19 pandemic affected their answers. A “yes/no” question assessed whether they or someone they knew was or is sick with COVID-19. These two questions were shown last and on a separate page to avoid any priming effect of the question.

## Results

### Initial Analyses

We first assessed whether participants felt that their answers were affected by the pandemic. A one-sample *t*-test against 0 revealed that participants believed that their judgments were affected by the pandemic to a significant degree (*M* = 2.60, *SD* = 2.18, 95% CI [2.40, 2.80]), *t*(453) = 25.40, *p* < 0.001, *d* = 1.19. A 2 (treatment of patients with COVID-19) × 2 (gender of the physician) × 4 (emotional expression) ANOVA revealed a significant main effect for treatment of patients with COVID-19, *F*(1, 438) = 68.35, *p* < 0.001, η*_*p*_*^2^ = 0.14, such that participants who saw a picture of a physician who supposedly treated patients with COVID-19 felt that their judgment was more strongly affected (*M* = 3.42, *SD* = 2.09, 95% CI [3.14, 3.68]) than those who saw a picture of a physician who did not (*M* = 1.82, *SD* = 1.98, 95% CI [1.55, 2.06]). A majority of participants (75.6%) reported that neither they nor someone they know in person was or is sick with COVID-19.

As expected, given media reports, a 2 (treatment of patients with COVID-19) × 2 (gender of the physician) × 4 (emotional expression) ANOVA on the perceived risk to the physician revealed a significant main effect for treatment of patients with COVID-19, *F*(1, 438) = 10.70, *p* < 0.001, η*_*p*_*^2^ = 0.04, such that physicians who supposedly treated patients with COVID-19 were perceived to be more at risk (*M* = 4.49, *SD* = 1.92, 95% CI [4.22, 4.74]) than those who did not (*M* = 3.68, *SD* = 2.08, 95% CI [3.44, 3.95]). Interestingly, the main effect of emotional expression also emerged significantly, *F*(3, 438) = 5.10, *p* = 0.002, η*_*p*_*^2^ = 0.03. Specifically, participants judged physicians to be less at risk when they showed happiness (*M* = 3.48, *SD* = 2.06, 95% CI [3.12, 3.84]) than when they showed sadness (*M* = 4.17, *SD* = 1.96, 95% CI [3.82, 4.59]), neutrality (*M* = 4.25, *SD* = 2.13, 95% CI [3.89, 4.58]), or anger (*M* = 4.41, *SD* = 1.88, 95% CI [4.06, 4.80]), which did not differ (detailed results of all *post hoc* tests appear in Supplementary Material).

Furthermore, as expected, a 2 (treatment of patients with COVID-19) × 2 (gender of the physician) × 4 (emotional expression) ANOVA on whether the work of physicians saved lives also revealed a significant main effect for treatment of patients with COVID-19, *F*(1, 438) = 33.44, *p* < 0.001, η*_*p*_*^2^ = 0.07, such that physicians who supposedly treated patients with COVID-19 were perceived to save more lives (*M* = 4.80, *SD* = 1.36, 95% CI [4.59, 4.95]) than those who did not (*M* = 4.06, *SD* = 1.48, 95% CI [3.86, 4.21]). Somewhat surprisingly, a significant main effect of emotional expression also emerged, *F*(3, 438) = 19.26, *p* < 0.001, η*_*p*_*^2^ = 0.12. Specifically, physicians who showed anger were perceived as saving significantly fewer lives (*M* = 3.69, *SD* = 1.68, 95% CI [4.59, 4.95]) than those who showed sadness (*M* = 4.20, *SD* = 1.52, 95% CI [4.59, 4.95]) who in turn were perceived as saving fewer lives than those who showed happiness (*M* = 4.83, *SD* = 1.17, 95% CI [4.59, 4.95]) or neutrality (*M* = 4.86, *SD* = 1.17, 95% CI [4.59, 4.95]) who did not differ significantly. This seems to suggest that showing a negative expression was seen as a sign of failure on the part of the physician independent of whether they treated patients with COVID-19 or not. This is somewhat at odds with findings that expressions of anger signal more competence than sad expressions (Tiedens, 2001).

### Person Perception

To assess the degree to which the perception of physicians was affected by the knowledge that they treat patients with COVID-19, the emotions they expressed, and their gender, we conducted a 2 (treatment of patients with COVID-19) × 2 (gender of the physician) × 4 (emotional expression) ANOVA on the positive evaluation composite variable. Only significant main effects of COVID-19 treatment, *F*(1, 438) = 7.97, *p* = 0.005, η*_*p*_*^2^ = 0.02, and emotional expression, *F*(3, 438) = 86.45, *p* < 0.001, η*_*p*_*^2^ = 0.37, emerged. Specifically, as predicted (H1) participants rated physicians who supposedly treated patients with COVID-19 more positively (*M* = 3.68, *SD* = 1.61, 95% CI [3.48; 3.82]) than those who did not (*M* = 3.33, *SD* = 1.63, 95% CI [3.14; 3.47]). As regards the impact of emotional expressions (H2), physicians who showed anger were rated least positively (*M* = 1.99, *SD* = 1.15, 95% CI [1.73, 2.22]) and those who showed happiness most positively (*M* = 4.71, *SD* = 0.95, 95% CI [4.47, 4.95]) with sadness (*M* = 3.40, *SD* = 1.50, 95% CI [3.17, 3.68]) and neutral expressions (*M* = 3.80, *SD* = 1.26, 95% CI [3.58, 4.03]) in between. All differences were significant. That is, contrary to our expectations (H3), emotional expressions did not interact with the information whether doctors doctors treated patients with COVID-19.

Furthermore, a 2 (treatment of patients with COVID-19) × 2 (gender of the physician) × 4 (emotional expression) ANOVA on whether participants would want to be treated by the physician shown if they needed treatment, also yielded significant main effects of treatment of patients with COVID-19, *F*(1, 438) = 8.73, *p* = 0.003, η*_*p*_*^2^ = 0.02, and emotional expression, *F*(3, 438) = 57.60, *p* < 0.001, η*_*p*_*^2^ = 0.28. A similar pattern emerged, in that participants preferred to be treated by the physician who supposedly treated patients with COVID-19 (*M* = 3.47, *SD* = 1.85, 95% CI [3.21, 3.62]) rather than the one who did not (*M* = 3.02, *SD* = 1.81, 95% CI [2.78, 3.18]). Furthermore, physicians who showed anger expressions were least preferred (*M* = 1.74, *SD* = 1.71, 95% CI [1.42, 2.01]) and those who showed happiness (*M* = 4.23, *SD* = 1.30, 95% CI [3.94, 4.51]) or neutrality (*M* = 3.88, *SD* = 1.44, 95% CI [3.60, 4.15]) were most preferred with sadness (*M* = 2.96, *SD* = 1.85, 95% CI [2.67, 3.28]) in between. All differences were significant (for details on *post hoc* tests, refer to Supplementary Material).

A very similar pattern emerged for the suggested salary. The 2 (treatment of patients with COVID-19) × 2 (gender of the physician) × 4 (emotional expression) ANOVA yielded again significant main effects for treatment of patients with COVID-19, *F*(1, 438) = 8.07, *p* = 0.005, η*_*p*_*^2^ = 0.02, and emotional expression, *F*(3, 438) = 11.33, *p* < 0.001, η*_*p*_*^2^ = 0.01. Physicians who were said to treat patients with COVID-19 were accorded a higher salary (*M* = 279274, *SD* = 34123, 95% CI [3.60, 4.15]) than those who did not (*M* = 270451, *SD* = 341233, 95% CI [266032; 274539]). Furthermore, physicians who showed anger (*M* = 259786, *SD* = 33287, 95% CI [253194; 265597]) were accorded a lower salary than those who showed sadness (*M* = 275172, *SD* = 34992, 95% CI [269085; 281926]), neutrality (*M* = 280383, *SD* = 34099, 95% CI [274679; 286110]), or happiness (*M* = 282627, *SD* = 29821, 95% CI [276547; 288673]), who did not differ significantly.

To assess whether the positive effects of the supposed treatment of patients with COVID-19 on the evaluation of the physicians or their suggested salary are due to the perception that these people put themselves at risk or the perception that they save lives or both of these plausible reasons, we conducted mediation analyses using Process 3.5 (Hayes, 2017). Table 1 shows the effect of the statistics.

**TABLE 1:** Results of mediation analyses for the judgments of physicians.

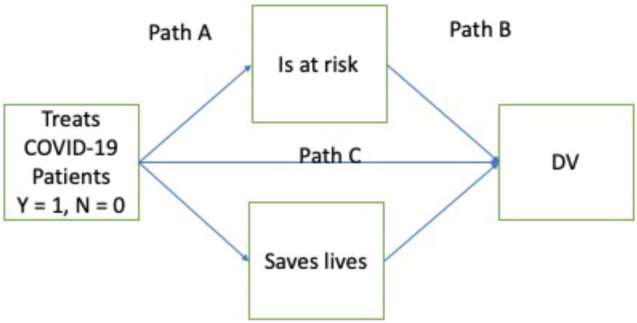							

	**Path A**		**Path B**			**Indirect effect**	
**DV**	**Risk**	**Save lives**	**Risk**	**Save lives**	**Path C**	**Risk**	**Save lives**
Positive evaluation	*b* = 0.81, *se* = 0.19 *t* = 4.30, *p* < 0.001	*b* = 0.74, *se* = 0.13 *t* = 5.56, *p* < 0.001	*b* = −0.10, *se* = 0.03 *t* = −3.30, *p* = 0.010	*b* = 0.64, *se* = 0.04 *t* = 14.62 *p* < 0.001	*b* = −0.04, *se* = 0.13 *t* = −0.30, *p* = 0.765	*b* = −0.08 *se* = 0.03 *LB* = −0.15 *UB* = −0.03	*b* = 0.48 *se* = 0.09 *LB* = 0.31 *UB* = 0.65
Salary	*b* = 0.81, *se* = 0.19 *t* = 4.30, *p* < 0.001	*b* = 0.74, *se* = 0.13 *t* = 5.56, *p* < 0.001	*b* = −37, *se* = 733 *t* = −0.05, *p* = 0.956	*b* = 9,469 *se* = 1,032 *t* = 9.17 *p* < 0.001	*b* = 1,828, *se* = 3,077 *t* = 0.59, *p* = 0.553	*b* = −30 *se* = 587 *LB* = −1,260 *UB* = 1,117	*b* = 7,025 *se* = 1,503 *LB* = 4,296 *UB* = 10,164
Treatment by this physician	*b* = 0.81, *se* = 0.19 *t* = 4.30, *p* < 0.001	*b* = 0.74, *se* = 0.13 *t* = 5.56, *p* < 0.001	*b* = −0.08, *se* = 0.04 *t* = −1.98, *p* = 0.47	*b* = 0.62 *se* = 0.05 *t* = 11.69 *p* < 0.001	*b* = 0.05, *se* = 0.16 *t* = 0.34, *p* = 0.733	*b* = −0.06 *se* = 0.03 *LB* = −0.13 *UB* = −0.00	*b* = 0.46 *se* = 0.09 *LB* = 0.29 *UB* = 0.64

As shown in Table 1, there is a strong positive indirect effect from treatment information to all three judgments *via* saving lives. The effect *via* risk is small in all cases and for salary non-significant. As such, the positive stance of participants toward the physician who treats patients with COVID-19 stems from the assumption that this person saves lives (and in fact more lives than other physicians). In contrast, the physician’s risk has no or a negative effect. This may suggest that participants consider the physician’s risk as a sign of incompetence rather than a fact that is linked to their professional situation.

No further main effects or interactions emerged significantly for any dependent variable.

## Discussion

Overall, as predicted, at the beginning of the pandemic the information that a physician treats patients with COVID-19 leads to a more positive evaluation both with regard to the evaluation of the physician as a caring and likable person and with regard to their desirability as a treating physician and even with regard to the salary they deserve. Yet, this positive evaluation was less due to the sacrifices made by these physicians in terms of exposing themselves to considerable risk at a time when treatments were not well-established, and no vaccine was available, but rather based on the utilitarian notion that they save lives. This stands in contradiction to the way media coverage portrayed the situation (Landauro, 2020). It is, therefore, important to further explore why the evaluations of participants of the physicians were almost unaffected by the fact that physicians treating patients with COVID-19 risked their lives. One possibility is that people believe that risks are an integral aspect of the work of physicians in a hospital due to the intense exposure to pathogens. Another possibility is, as suggested earlier, that people acknowledge that not only the work of physicians involves risks but also physicians should be able to avoid them. Accordingly, as much as this is not the case, this is the fault of physicians and as such not something that deserves appreciation.

Notably, the positive evaluations of physicians treating patients with COVID-19 were found for both male and female physicians and were independent of the emotional expressions the physicians showed. The latter finding was somewhat unexpected. As noted earlier, emotional expressions are perceived as social signals that tell others something about the person and the situation (Hess and Hareli, 2019). In fact, the situational information provided by an emotional expression is often dominant over character information as it is more proximal to the evaluation (Küster et al., 2019). However, in this study, this was only inconsistently the case. This is especially surprising as it was made clear in the instructions that the emotional expressions were shown in the context of physicians discussing their situation at work.

Specifically, participants preferred being treated by a happy rather than an angry physician and evaluated a happy physician more positively. These findings are in line with research showing that happy people are often evaluated more positively on a wide variety of traits (Li et al., 2021). Yet, for these ratings, there is no evidence that participants related the expressions to the situation.

For example, anger in the context of treating patients with COVID-19 could also be a sign of caring and hence lead to a positive evaluation (Hareli and Hess, 2010), yet this was not found. By contrast, the emotion effect on risk assessment suggests that the expression was taken into account when thinking about the effects of the pandemic as participants considered those who showed happiness to be less at risk. Since happiness typically signals that all is well (Scherer, 1987), this would make sense.

Yet again, the emotion effect did not interact with the information on treatment of patients with COVID-19. In this sense, it seemed that treating patients with COVID-19 remained a rather abstract notion for the participants, a behavior that they considered positive because it saves lives, but that in the end did not inform about the physician as a person. This information was preferentially taken from the emotional expressions shown. This notion is also supported by the fact that the effects of emotional expression were generally larger than those of knowledge about treatment of patients with COVID-19. As such, it may not be a surprise that over time the admiration of these doctors has waned in the press.

Yet, it is also possible that despite the fact that participants were told that the emotions that the physicians expressed were a response to the situation, it was still not sufficiently clear what these emotions were about. Maybe the information that the objects of anger or sadness, for example, were the individuals who did not obey medical advice expected to reduce their chances to catch COVID-19, would have affected the judgments of physicians treating patients with COVID-19 differently. This is one limitation of this study. Alternatively, it is possible that judgments based on social stereotypes such as the notion that physicians do not react emotionally to their job are quite robust, leading participants to disregard this information. Future research could, for example, use vignettes to more clearly situate the physicians in a social context that elicits their emotions.

However, the results of our study clearly show that the emotions that physicians express about their work affect how they are perceived. Furthermore, we were able to show that the current pandemic affected to way physicians who treated patients with COVID-19 were perceived by the public.

## Data Availability Statement

The datasets presented in this study can be found in online repositories. The names of the repository/repositories and accession number(s) can be found below: https://osf.io/de8f3/?view_only=3fed6b0dff7943e4ac5f948a9a6ea800.

## Ethics Statement

The studies involving human participants were reviewed and approved by IRB of the Faculty of Social Sciences of the University of Haifa (IRB Approval No. 347/20). The patients/participants provided their written informed consent to participate in this study.

## Author Contributions

SH, UH, and FB designed the study and wrote the manuscript. OD helped with the literature review. SH and OD programmed the studies and prepared the materials. All authors were involved in the data analysis.

## Conflict of Interest

The authors declare that the research was conducted in the absence of any commercial or financial relationships that could be construed as a potential conflict of interest.

## Publisher’s Note

All claims expressed in this article are solely those of the authors and do not necessarily represent those of their affiliated organizations, or those of the publisher, the editors and the reviewers. Any product that may be evaluated in this article, or claim that may be made by its manufacturer, is not guaranteed or endorsed by the publisher.
