# Supplementary material for: Does It Pay to Treat Patients With Coronavirus Disease 2019? Social Perception of Physicians Treating Patients With Coronavirus Disease 2019
Source: Front Psychol. 2022 Jan 13;12:781220. doi: 10.3389/fpsyg.2021.781220 (PMC8792981; doi:10.3389/fpsyg.2021.781220)
Supplement: Supplementary file 1 [file Table_1.DOCX]

Results of Post Hoc analyses

Table S1

Effect of emotion on perception of risk

| **Post Hoc Comparisons - Emotion** | | | | | | | | | | | |
| --- | --- | --- | --- | --- | --- | --- | --- | --- | --- | --- | --- |
|  | |  | | **Mean Difference** | | **SE** | | **t** | | **p _tukey_** | |
| Neutral |  | Sadness |  | 0.029 |  | 0.262 |  | 0.112 |  | 0.999 |  |
|  |  | Anger |  | -0.198 |  | 0.257 |  | -0.771 |  | 0.867 |  |
|  |  | Happiness |  | 0.757 |  | 0.254 |  | 2.981 |  | 0.016 |  |
| Sadness |  | Anger |  | -0.228 |  | 0.272 |  | -0.836 |  | 0.837 |  |
|  |  | Happiness |  | 0.728 |  | 0.269 |  | 2.703 |  | 0.036 |  |
| Anger |  | Happiness |  | 0.955 |  | 0.264 |  | 3.613 |  | 0.002 |  |
|  | | | | | | | | | | | |
| *Note.*  P-value adjusted for comparing a family of 4 | | | | | | | | | | | |
|  | | | | | | | | | | | |

Table S2

Effect of emotion on perception of the physician as saving patients’ lives

| **Post Hoc Comparisons - Emotion** | | | | | | | | | | | |
| --- | --- | --- | --- | --- | --- | --- | --- | --- | --- | --- | --- |
|  | |  | | **Mean Difference** | | **SE** | | **t** | | **p _tukey_** | |
| Neutral |  | Sadness |  | 0.636 |  | 0.179 |  | 3.553 |  | 0.002 |  |
|  |  | Anger |  | 1.157 |  | 0.176 |  | 6.588 |  | < .001 |  |
|  |  | Happiness |  | 0.018 |  | 0.174 |  | 0.102 |  | 1.000 |  |
| Sadness |  | Anger |  | 0.521 |  | 0.186 |  | 2.803 |  | 0.027 |  |
|  |  | Happiness |  | -0.619 |  | 0.184 |  | -3.362 |  | 0.005 |  |
| Anger |  | Happiness |  | -1.140 |  | 0.181 |  | -6.308 |  | < .001 |  |
|  | | | | | | | | | | | |
| *Note.*  P-value adjusted for comparing a family of 4 | | | | | | | | | | | |

Table S3

Effect of emotion on positive evaluation of the physician as saving patients’ lives

| **Post Hoc Comparisons - Emotion** | | | | | | | | | | | | |
| --- | --- | --- | --- | --- | --- | --- | --- | --- | --- | --- | --- | --- |
|  | |  | | **Mean Difference** | | **SE** | | **t** | | | **p _tukey_** | |
| Neutral |  | Sadness |  | 0.375 |  | 0.172 |  | 2.187 | |  | 0.128 |  |
|  |  | Anger |  | 1.828 |  | 0.168 |  | 10.856 | |  | < .001 |  |
|  |  | Happiness |  | -0.910 |  | 0.166 |  | -5.467 | |  | < .001 |  |
| Sadness |  | Anger |  | 1.453 |  | 0.178 |  | 8.151 | |  | < .001 |  |
|  |  | Happiness |  | -1.285 |  | 0.176 |  | -7.287 | |  | < .001 |  |
| Anger |  | Happiness |  | -2.738 |  | 0.173 |  | -15.809 | |  | < .001 |  |
|  | | | | | | | | | | | | |
| *Note.*  P-value adjusted for comparing a family of 4 | | | | | | | | |  |  |  |  |

Table S4

Effect of emotion on wanting to be treated by the physician

| **Post Hoc Comparisons - Emotion** | | | | | | | | | | | |
| --- | --- | --- | --- | --- | --- | --- | --- | --- | --- | --- | --- |
|  | |  | | **Mean Difference** | | **SE** | | **t** | | **p _tukey_** | |
| Neutral |  | Sadness |  | 0.900 |  | 0.208 |  | 4.333 |  | < .001 |  |
|  |  | Anger |  | 2.161 |  | 0.204 |  | 10.598 |  | < .001 |  |
|  |  | Happiness |  | -0.350 |  | 0.201 |  | -1.739 |  | 0.305 |  |
| Sadness |  | Anger |  | 1.260 |  | 0.216 |  | 5.840 |  | < .001 |  |
|  |  | Happiness |  | -1.251 |  | 0.213 |  | -5.859 |  | < .001 |  |
| Anger |  | Happiness |  | -2.511 |  | 0.210 |  | -11.976 |  | < .001 |  |
|  | | | | | | | | | | | |
| *Note.*  P-value adjusted for comparing a family of 4 | | | | | | | | | | | |
| Table S5  Suggested physicians’ salary as a function of emotion   \| **Post Hoc Comparisons - Emotion** \| \| \| \| \| \| \| \| \| \| \| \| \| --- \| --- \| --- \| --- \| --- \| --- \| --- \| --- \| --- \| --- \| --- \| --- \| \|  \| \|  \| \| **Mean Difference** \| \| **SE** \| \| **t** \| \| **p _tukey_** \| \| \| Neutral \|  \| Sadness \|  \| 4889.185 \|  \| 4373.750 \|  \| 1.118 \|  \| 0.679 \|  \| \|  \|  \| Anger \|  \| 20999.435 \|  \| 4291.099 \|  \| 4.894 \|  \| < .001 \|  \| \|  \|  \| Happiness \|  \| -2215.454 \|  \| 4239.688 \|  \| -0.523 \|  \| 0.954 \|  \| \| Sadness \|  \| Anger \|  \| 16110.250 \|  \| 4541.872 \|  \| 3.547 \|  \| 0.002 \|  \| \|  \|  \| Happiness \|  \| -7104.639 \|  \| 4493.331 \|  \| -1.581 \|  \| 0.390 \|  \| \| Anger \|  \| Happiness \|  \| -23214.889 \|  \| 4412.921 \|  \| -5.261 \|  \| < .001 \|  \| \|  \| \| \| \| \| \| \| \| \| \| \| \| \| *Note.*  P-value adjusted for comparing a family of 4 \| \| \| \| \| \| \| \| \| \| \| \| \|  \| \| \| \| \| \| \| \| \| \| \| \| | | | | | | | | | | | |
